# Supplementary material for: Splice-Junction-Based Mapping of Alternative Isoforms in the Human Proteome
Source: Cell Rep. Author manuscript; Available in PMC 2020 Jan 15. (PMC6961840; doi:10.1016/j.celrep.2019.11.026)
Supplement: 2 [file NIHMS1546469-supplement-2.zip › DF1/PXD010154/Heart/Heart_4_MYBPC3_RTDSHEDTGILDFSSLLK.pdf]

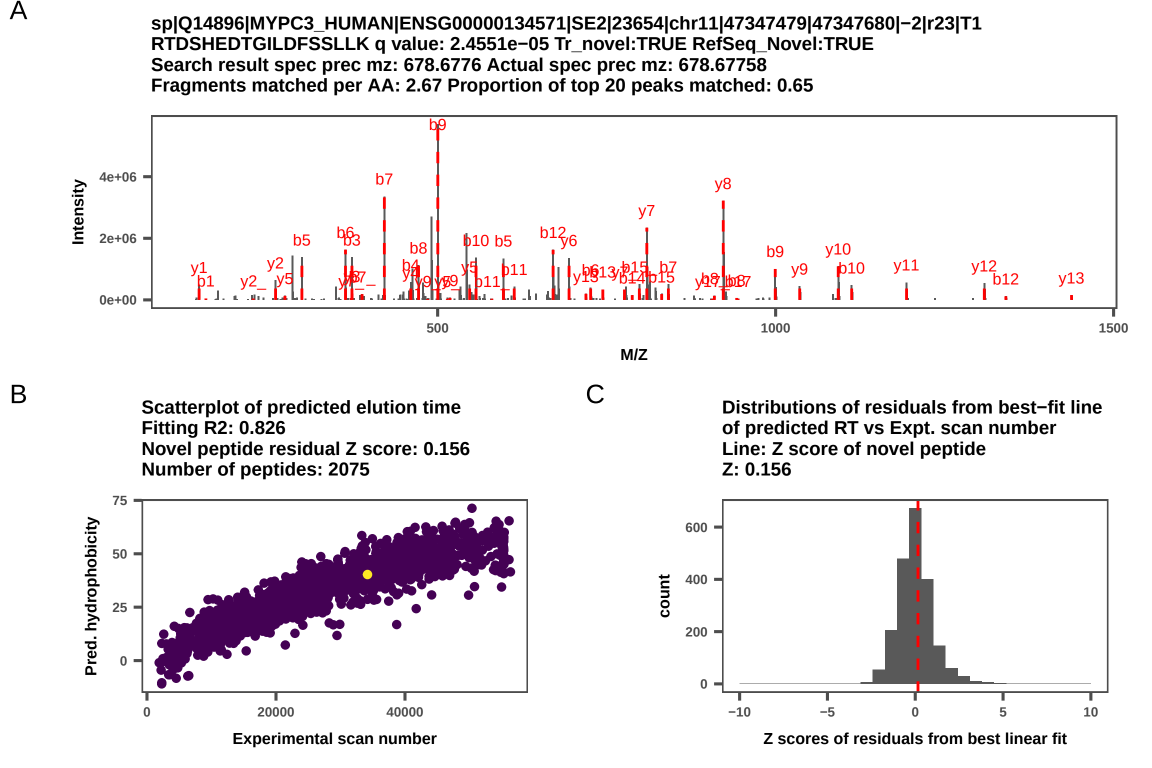
A
sp|Q14896|MYPC3_HUMAN|ENSG00000134571|SE2|23654|chr11|47347479|47347680|−2|r23|T1
RTDSHEDTGILDFSSLLK q value: 2.4551e−05 Tr_novel:TRUE RefSeq_Novel:TRUE
Search result spec prec mz: 678.6776 Actual spec prec mz: 678.67758
Fragments matched per AA: 2.67 Proportion of top 20 peaks matched: 0.65
Intensity
4e+06
2e+06
0e+00
M/Z
500
1000
1500
b9
b7
y8
y7
b5
b6
b3
b8
b10
b12
y6
y10
y11
y12
y13
b12
y1
b1
y2
B
Scatterplot of predicted elution time
Fitting R2: 0.826
Novel peptide residual Z score: 0.156
Number of peptides: 2075
Pred. hydrophobicity
Experimental scan number
75
50
25
0
20000
40000
C
Distributions of residuals from best−fit line
of predicted RT vs Expt. scan number
Line: Z score of novel peptide
Z: 0.156
count
600
400
200
0
−10
−5
0
5
10
Z scores of residuals from best linear fit
